# Development, cross-validation and greenness assessment of capillary electrophoresis method for determination of ALP in pharmaceutical dosage forms – an alternative to liquid chromatography†

**DOI:** 10.1039/d4ra05715e

**Published:** 2024-10-18

**Authors:** Zvonimir Mlinarić, Lu Turković, Ivor Babić, Tajana Silovski, Nina Kočevar Glavač, Miranda Sertić

**Affiliations:** a University of Zagreb Faculty of Pharmacy and Biochemistry, Department of Pharmaceutical Analysis Ante Kovačića 1 10000 Zagreb Croatia miranda.sertic@pharma.unizg.hr; b University Hospital Centre Zagreb, Department of Oncology Kišpatićeva 12 10000 Zagreb Croatia; c University of Zagreb School of Medicine Šalata 2 10000 Zagreb Croatia; d University of Ljubljana, Faculty of Pharmacy, Department of Pharmaceutical Biology Aškerčeva cesta 7 1000 Ljubljana Slovenia

## Abstract

Breast cancer treatment has made tremendous progress in recent years and new therapies are emerging continuously. Alpelisib (ALP) is a novel phosphoinositide-3-kinase (PI3K) inhibitor recently approved for human receptor-positive, human epidermal growth factor receptor 2-negative, PIK3CA-mutated metastatic breast cancer in combination with fulvestrant. ALP has been the subject of only a limited number of preclinical *in vitro* and *in vivo* studies using different chromatographic techniques. However, no research has been published on analyzing ALP using capillary electrophoresis (CE). The absence of pharmacopoeial monographs for ALP in both the European and United States Pharmacopoeias highlights the urgent need to develop a reliable analytical method for its quality control in both industry and regulatory authorities. In this work, we have developed a first-ever CE method for the determination of ALP in pharmaceutical dosage forms in just 1.4 minutes. This was achieved with a 25 mM borate buffer at pH 9.3, 30 kV separation voltage and 30 °C capillary temperature. The proposed method was validated according to the ICH guidelines regarding selectivity, linearity (*r* = 0.9988), precision (RSD < 5.9%), accuracy (bias < 3.0%) and robustness (RSD < 3.2%). It was applied to the pharmaceutical dosage form of ALP and was shown to be suitable for the reliable determination of ALP. Furthermore, to demonstrate the applicability of the CE as an alternative technique to more commonly used HPLC in the analysis of drugs, cross-validation of CE and HPLC methods was performed. Bland–Altman analysis showed that the average difference in determined concentrations between CE and HPLC over a range of 10–100 μg mL^−1^ was 0.87 μg mL^−1^ (*p* = 0.6390, *N* = 19) meaning that there is no difference in the performance of CE and HPLC in the determination of ALP in pharmaceutical dosage forms. The environmental impact of both methods was assessed using AGREE software and scores for CE and HPLC were calculated to be 0.74 and 0.51, respectively. Because of equally reliable analytical performance and greener analysis, CE should be considered as an alternative technique to HPLC in the analysis of ALP pharmaceutical dosage forms.

## Abstract 2

Novel CE and HPLC methods for the determination of ALP in pharmaceutical dosage forms were developed, validated according to ICH guidelines, applied to real pharmaceutical samples, cross-validated and greenness assessment was performed.

## Graphical abstract

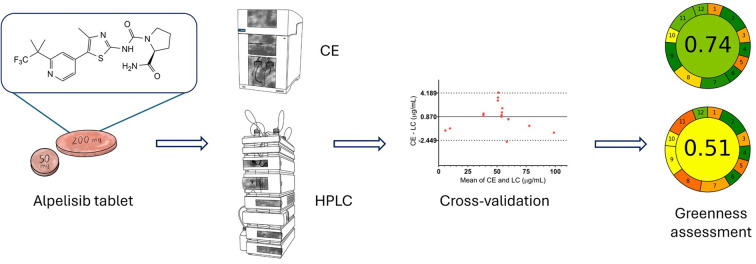

## Introduction

According to the World Health Organization (WHO), around 2.3 million cases of breast cancer are diagnosed each year, and around 685 thousand patients die from it worldwide.^1^ This makes breast cancer the leading cause of cancer deaths in women in the world. The expected global burden of cancer by 2040 is 28.4 million cases which represents a 47% rise from 2020.^1^ Typical breast cancer treatment combines surgery, radiation, and systemic treatment; the latter encompasses endocrine therapy, chemotherapy, and targeted therapy including immunotherapy.^2^ Breast cancer medication has made tremendous progress in recent decades, and new medicines continue to enter the market. Alpelisib (ALP) is a novel targeted breast cancer drug which inhibits phosphoinositide 3-kinase (PI3K). It was first approved by the FDA in 2019, in combination with fulvestrant, for the treatment of hormone receptor-positive (HR+), human epidermal growth factor receptor 2-negative (HER2−), PIK3CA-mutated, advanced or metastatic breast cancer, and has been available in the EU since EMA's marketing authorization in 2020.^3^

The development of new drugs is intrinsically linked to the development of analytical methods for the determination of pharmacokinetic properties and quality control. ALP has been the subject of only a limited number of preclinical *in vitro* and *in vivo* studies using different chromatographic techniques; high-performance liquid chromatography with fluorescence detection (HPLC-FLD) for the study of ALP metabolism and pharmacokinetics in rats,^4^ and ultra-performance liquid chromatography coupled with tandem mass spectrometry (UPLC-MS/MS) for the determination of ALP in mice and human plasma.^5,6^ There is one stability-indicating method employing liquid chromatography-quadrupole-time-of-flight mass spectrometry (LC-QTOF-MS/MS) for structural characterization of the forced degradation products of ALP.^7^ Few electrochemical methods for the analysis of ALP in biological fluids and tablets were reported, but it was not specified which exact formulation was analysed.^8–10^ However, no research has been published on the analysis of ALP by capillary electrophoresis (CE). The fact that no pharmacopoeial monographs for ALP are available in either the European or the United States Pharmacopoeia,^11,12^ shows the urgent need for the development of a reliable analytical method for the quality control of ALP.

CE has been recognized as an alternative or complementary analytical tool with specific advantages over chromatographic techniques.^13^ Of these, a considerable advantage is the suitability of the application to a wide range of analytes, from organic and inorganic, charged and neutral, water-soluble and insoluble, polar and nonpolar, small-sized and polymeric, to aromatic and aliphatic molecules. In addition, CE requires small sample and solvent volumes and enables faster analysis times.^14,15^

Along with a need to develop reliable, efficient, and cost-effective methods, sustainability is gaining increased attention as a growing element in green analytical chemistry. Indeed, CE has been proven to meet the criteria of a sustainable and eco-friendly analytical solution due to its significant advantages, both at a level of environmental compatibility and analytical performance.^16,17^

Based on the background mentioned above, ALP as an active pharmaceutical ingredient is an attractive candidate for the development of a CE analytical method for use in pharmacological research and pharmaceutical quality control. As this area has not been investigated so far, our work aimed to develop and validate a new CE method that will allow the quantitative determination of ALP in finished pharmaceutical products, as well as compare the proposed method to an HPLC method – a complementary analytical technique and a golden standard in the pharmaceutical industry. Additional focus will be given to the evaluation of method greenness according to selected guidelines.

The ultimate goal of our work is to support the development of the analytical determination of ALP by industry and regulatory authorities, by providing a reliable and sustainable CE method that enables the quantitative determination of ALP in finished pharmaceutical products.

## Experimental

### Instrumentation

CE experiments were carried out on High-Performance Capillary Electrophoresis G1600A (Agilent Technologies, Santa Clara, CA, USA) with diode array detection. Data were collected and analyzed using ^3D^CE/MSD ChemStation (Agilent Technologies, Santa Clara, CA, USA). An uncoated fused-silica capillary with a 50 μm internal diameter, total length of 37 cm, and effective length of 28.5 cm was used (Agilent Technologies, Santa Clara, CA, USA).

For the HPLC analysis, an Agilent 1100 system equipped with a diode array detector was used (Agilent Technologies, Santa Clara, CA, USA). Data were collected and analyzed using ChemStation for LC 3D systems (Agilent Technologies, Santa Clara, CA, USA). Microsoft Office 365 Excel (Redmond, WA, USA) and GraphPad Prism 8 (Boston, MA, USA) were used for data analysis and visualisation.

### Chemicals

HPLC-grade methanol (MeOH) was purchased from Carlo Erba (Milan, Italy). Sodium dodecyl sulfate (SDS) was obtained from MilliporeSigma (Burlington, MA, USA), 1 M sodium hydroxide solution and 50 mM sodium tetraborate buffer solution pH 9.3 for CE were purchased from Agilent Technologies (Santa Clara, CA, USA). Ultrapure water, used throughout this work, was purified using a Merck Millipore Milli-Q IQ 7015 system (Burlington, MA, USA). The standard of ALP (purity > 98%) was obtained from MedChemExpress (Princeton, NJ, USA). The chemical structure of ALP is shown in Fig. S1.† 4-Aminobenzoic acid (internal standard, IS) was from Fluka (Buchs, Switzerland). Piqray 50 and 200 mg film-coated tablets were obtained from Novartis (Basel, Switzerland).

### Experimental conditions

#### Standard solutions

Standard solutions of ALP and IS were prepared at 0.5 mg mL^−1^ in MeOH and stored at +4 °C. The stock solutions were stable for at least two months. Working solutions for the analyses were prepared in 50% MeOH at 5, 10, 40, 50, 60, 75, and 100 μg mL^−1^ concentration levels.

#### Sample preparation of ALP tablets

The amount of ALP tablet powder containing 2.5 mg ALP was weighed in a 10 mL volumetric flask. Afterwards, IS (2.5 mg) and MeOH (7 mL) were added, and the mixture was vortex-mixed for 5 min, followed by ultrasonication for 30 min. The volume was filled up to the mark and the aliquots of the solution were centrifuged for 3 min at 10 000 rpm. The obtained supernatants were diluted five-fold into 50% MeOH for the analysis.

#### CE conditions

**Fig. 1:**
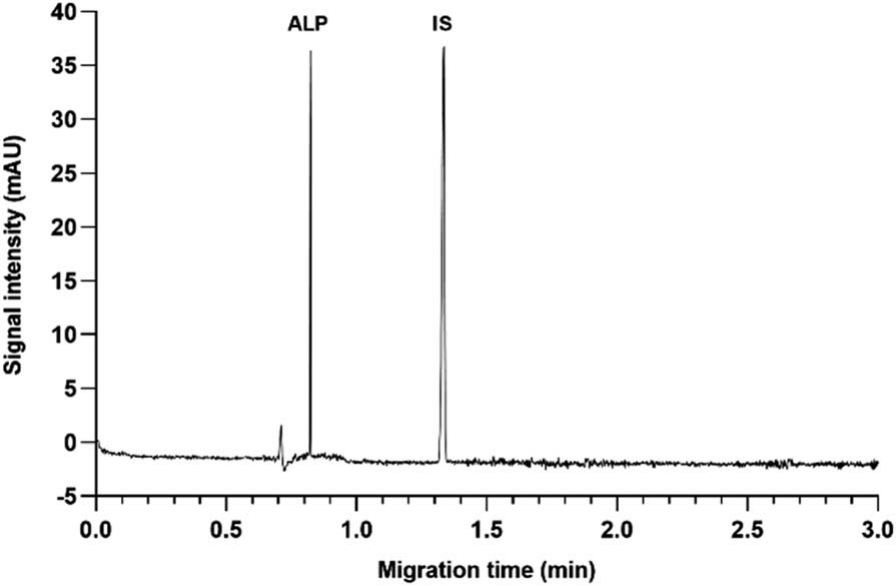
CZE electropherogram of ALP and IS standard solutions at 50 μg mL^−1^, 25 mM borate at pH 9.3, 30 kV, 30 °C.

Before the first use, a new capillary was conditioned with methanol for 10 min, followed by 1 M NaOH for 10 min, water for 10 min, and the running buffer for 20 min. Daily conditioning was done by flushing with 0.1 M NaOH for 10 min, water for 10 min, and the running buffer for 10 min. At the end of the working day, the capillary was washed with water for 20 minutes and stored in water vials until the next usage. Running buffers were changed after every 10 runs. All liquids were filtered through a 0.2 μm PES filter before use. Method run time was set at 3 min, with 3 min preconditioning with the running buffer, and 2 min postconditioning with 0.1 M NaOH. Samples were injected for 6 seconds under 50 mbar pressure. The optimal running buffer composition was 25 mM sodium borate buffer pH 9.3. The analyses were performed at 30 kV capillary voltage and 30 °C cassette temperature. The analyte and the IS were detected at 216 nm. A representative electropherogram of ALP and IS at the optimal conditions is shown in Fig. 1.

#### HPLC conditions

**Fig. 2:**
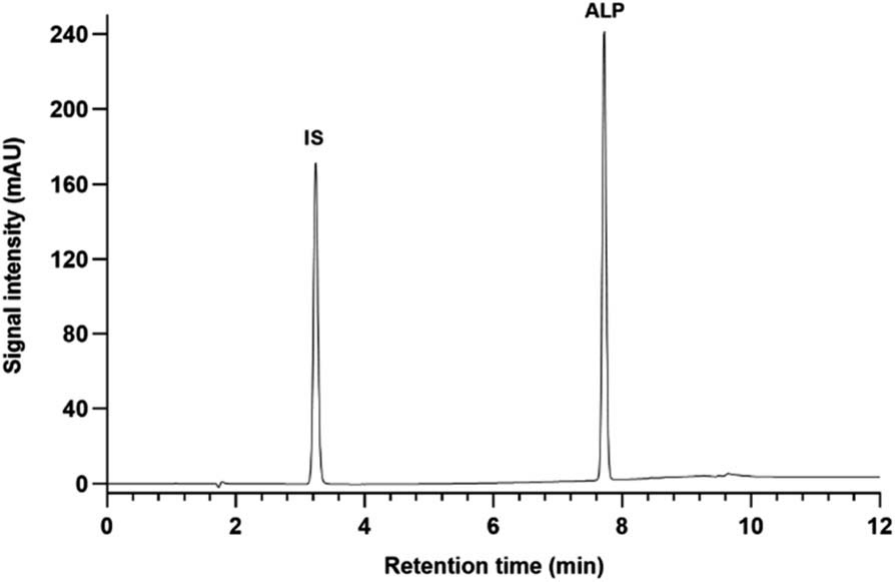
Chromatogram of ALP and IS standard solutions at 50 μg mL^−1^, detection at 315 nm.

A Gemini C18 column, dimensions 150 × 4.6 mm, particle size 5 μm (Phenomenex, Torrance, CA, USA), thermostated at 25 °C was used for separation. The mobile phase consisted of ultrapure water with 0.1% formic acid (mobile phase A) and methanol containing 0.1% formic acid (mobile phase B) in gradient elution at 1 mL min^−1^ flow rate. The total runtime was 16 min. The gradient composition is depicted in Table 1. The analyte and IS were detected at 315 nm wavelength. A representative chromatogram of ALP and IS is shown in Fig. 2.

**Table 1:** Gradient table for the developed HPLC method

Time (min)	Mobile phase B%
0	30
7	100
10	100
10.5	30
16	30

## Results and discussion

### CE method development

The first step of every CE method development is the choice of the CE mode, and the pH of the running buffer. Since ALP is a basic molecule, capillary zone electrophoresis (CZE) in basic pH was the technique of choice to ensure high electrophoretic mobility of ALP. This would also secure strong electroosmotic flow due to highly deprotonated silanol groups on the inner capillary wall. The preliminary results demonstrated that the routinely used borate buffer of pH 9.3 enabled fast analysis of ALP in CZE mode.

**Fig. 3:**
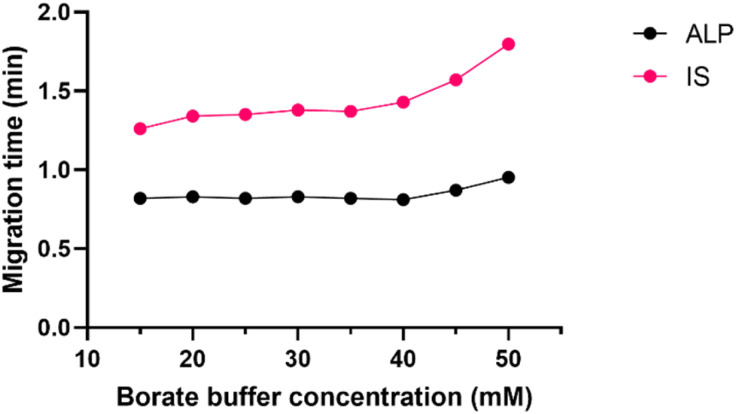
Effect of borate buffer concentration (15–50 mM) on ALP and IS migration times, pH 9.3, 30 °C, 30 kV.

Borate buffer concentration was investigated in the range of 15 to 50 mM. This is a pivotal step in developing the CZE method since the buffer concentration directly influences the electroosmotic flow (EOF) and the reproducibility of the separation system. Higher buffer concentration decreases EOF and prolongs the analysis. It may also generate a high electric current within the capillary causing Joule heating. On the other hand, if the buffer concentration is too low, the sample might adsorb to the capillary surface. Finally, if the buffer and the sample conductivity differ, the analyte peak shape distorts. At all tested buffer concentrations, the peak shape and symmetry were adequate, and as seen in Fig. 3 the migration time did not change much until the concentration of 45 mM was investigated. To avoid the aforementioned analyte adsorption and Joule heating, the 25 mM borate buffer was chosen for the subsequent experiments.

Although ALP analysis is already possible using CZE, since this is the first report of a capillary electrophoretic determination of this drug, the influence of background electrolyte (BGE) additions was also investigated. The addition of SDS, as the most commonly used surfactant, was tested in the concentrations 20–30 mM. Since this changes the CE mode to micellar electrokinetic chromatography (MEKC), the observed increase in the ALP migration times and total analysis time is not surprising. However, the total analysis time was not too long (Fig. S2†), and such BGE could also be used for the analysis of ALP in the presence of other neutral analytes. It is worth mentioning that the migration order of ALP and the IS is reversed in MEKC.

A similar effect of the increase of migration times was observed with the addition of the organic solvent to the borate buffer. Namely, the addition of the organic solvent changes the electrolyte viscosity of the BGE. Its effects to the EOF are complex, they may alter the selectivity of the method and have to be determined experimentally. The addition of 10% methanol did not result in an improved peak shape, had no influence on the selectivity of the method, and had little influence on the resolution between ALP and IS (Fig. S3†). Moreover, it slightly prolonged the analysis time. Since the aim was to keep the method as eco-friendly as possible and no improvements were noticed by the addition of surfactants or organic solvent to the BGE, only 25 mM borate buffer pH 9.3 was used as optimal BGE composition.

**Fig. 4:**
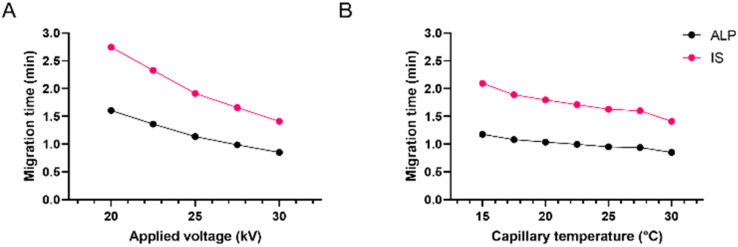
Effect of (A) applied voltage and (B) capillary temperature on ALP and IS migration times; 25 mM borate at pH 9.3.

Finally, for fine-tuning, the influences of capillary temperature and separation voltage on the analysis time, peak shape, and resolution were investigated. The separation voltage was tested in the range of 15 to 30 kV. This parameter, directly influencing the strength of the electric field, showed the strongest effect on the migration times (Fig. 4A). As expected, the fastest analysis was obtained at 30 kV. Before it was chosen as optimal, the influence on the peak area, resolution, and current was also investigated. Although the increase of separation voltage decreases peak area, the method's sensitivity remained satisfactory in the entire range. The resolution between ALP and the IS remained extremely high due to very narrow peaks and short migration times. Finally, the applied 30 kV voltage resulted in a current of around 110 μA, posing little risk of Joule heating.

Capillary temperature was investigated in the range of 15 to 30 °C. In Fig. 4B it can be observed that the migration times increased by decreasing the capillary temperature. This is in accordance with the known influence of temperature on the viscosity of the BGE, which affects both the EOF and the electrophoretic mobility of the analyte. Since the peak shape and the resolution were satisfactory in the entire tested range, the preference was given to 30 °C since the analysis time was the shortest, the peaks were narrow and symmetrical, and the resolution was excellent. Due to low buffer concentration in the BGE, even at higher voltages, the current within the capillary was not high with little risk of Joule heating.

### CE method validation

The newly developed CE method was validated according to the ICH Q2(R2) guidelines in terms of selectivity, linearity, limits of detection and quantification, precision and accuracy, and robustness.^18^ The peak area of ALP was corrected with the IS.

The linearity of the proposed method was evaluated in the range 10–100 μg mL^−1^ with the obtained correlation coefficient of 0.9988.

Selectivity was assessed as peak purity from 200 to 400 nm. The obtained scores ranged from 998.9 to 1000.0, demonstrating that ALP and IS were not co-migrating mutually nor with any other compounds.

The precision of the method was expressed as repeatability and intermediate precision. Repeatability was assessed by 6 measurements on 3 concentration levels within a single day, while intermediate precision was evaluated by 3 measurements on 3 concentration levels for 3 days, totalling 9 measurements per concentration level. The accuracy of the method was expressed as the bias of measured concentrations from the nominal values at 3 concentration levels. Results are shown in Table 2.

**Table 2:** Accuracy (*N* = 3) and precision results – repeatability (*N* = 6 samples per concentration level in one day) and intermediate precision (*N* = 9 samples per concentration level in 3 days)

ALP concentration (μg mL^−1^)	Accuracy	Repeatability		Intermediate precision	
	Bias (%)	Migration time RSD (%)	IS-corrected peak area RSD (%)	Migration time RSD (%)	IS-corrected peak area RSD (%)
40	3.0	0.08	1.25	1.73	4.96
50	0.9	0.17	0.67	1.00	3.93
60	2.3	0.29	1.10	0.31	5.87

Traditionally, the robustness is a weak point of the CE technique since it is usually less robust than the HPLC technique. Therefore, robustness of the developed CE method was assessed by slightly varying the BGE concentrations, capillary temperature and separation voltage and it was expressed as the bias of the IS-corrected peak area and the RSD of the migration times (Table 3).

**Table 3:** Robustness results (*N* = 6 samples per condition (3 positive, 3 negative))

Parameter	Migration time RSD (%)	IS-corrected peak area RSD (%)
BGE concentration (25 ± 1 mM)	0.70	1.53
Capillary temperature (30 ± 1 °C)	1.11	1.51
Applied voltage (30–1 kV)	4.26	3.19

Since all validation parameters were in acceptable ranges, the developed method is successfully validated for the intended use.

### Analysis of ALP tablets

The developed and validated CE method was applied to the pharmaceutical dosage form of ALP (Piqray 50 mg film-coated tablets) to determine its content. Found concentrations of ALP were in agreement with the labelled content, and the mean recovery of ALP was 98.7 ± 1.2%.

Next, both CE and HPLC methods were applied to another pharmaceutical dosage form of ALP (Piqray 200 mg film-coated tablets) to test whether there were any differences in their applicability to the analysis of ALP in real samples (Fig. S4–S5†). No interferences of excipients with the analyte's peak were observed. Both methods detected similar concentrations of ALP in the same sample with relative concentrations (CE to HPLC) ranging from 99.7 to 102.1%.

### HPLC method validation, cross-validation

To further demonstrate the applicability of the proposed method in the pharmaceutical industry or regulatory setting, cross-validation between the CE and HPLC as a golden standard was conducted in terms of precision, accuracy, and linearity. The results are presented in Tables 4 and 5. The precision and accuracy of both methods were acceptable due to low RSD and bias values, respectively. Furthermore, the validation parameters of CE and HPLC were comparable meaning that both methods could be applied for the intended purpose.

**Table 4:** Precision and accuracy of the CE and HPLC method

ALP concentration (μg mL^−1^)	CE		HPLC	
	RSD (%)	Bias (%)	RSD (%)	Bias (%)
40	0.4	−2.6	0.2	−4.6
50	0.9	5.5	0.3	−0.1
60	2.9	−1.6	0.4	1.4

**Table 5:** Summary of key cross-validation results for the CE and LC methods

Validation parameter	CE	HPLC
Linear range	10–100 μg mL^−1^	10–100 μg mL^−1^
Weighting factor	1/*x*^2^	1/*x*^2^
Slope	0.00414	0.0253
Intercept	0.0001096	−0.0281
Correlation coefficient	0.9988	0.9988
Detection limit^a^ (μg mL^−1^)	1.67	0.06
Quantitation limit^b^ (μg mL^−1^)	5.00	0.20

a Calculated as the concentration of the analyte at which the signal-to-noise ratio is 3 : 1.

b Calculated as the concentration of the analyte at which the signal-to-noise ratio is 10 : 1.

**Fig. 5:**
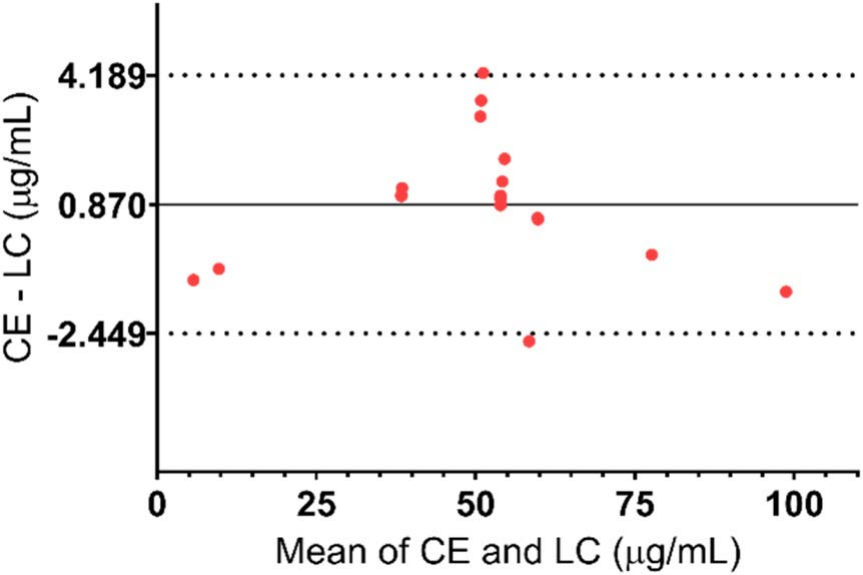
Bland–Altman plot of the results obtained with both methods (difference of measured concentrations with CE and LC methods *vs.* average value of both methods). Bias (the average of the differences) = 0.870; 95% confidence interval = −2.449–4.189 μg mL^−1^*p*-value (paired *t*-test): 0.6390, *N* = 19.

The same samples including calibration standards (*N* = 7), quality controls (*N* = 6), and ALP dosage form extracts (*N* = 6) were analysed simultaneously using both methods and the results were compared using a Bland–Altman plot, as shown in Fig. 5. Differences between the results for all the samples were less than variation during the validation of the methods. Additionally, the 95% confidence interval is narrow which further emphasizes small differences in CE and HPLC methods. Therefore, it can be concluded that both methods are valid for the determination of ALP in its dosage form.

### Greenness assessment

**Fig. 6:**
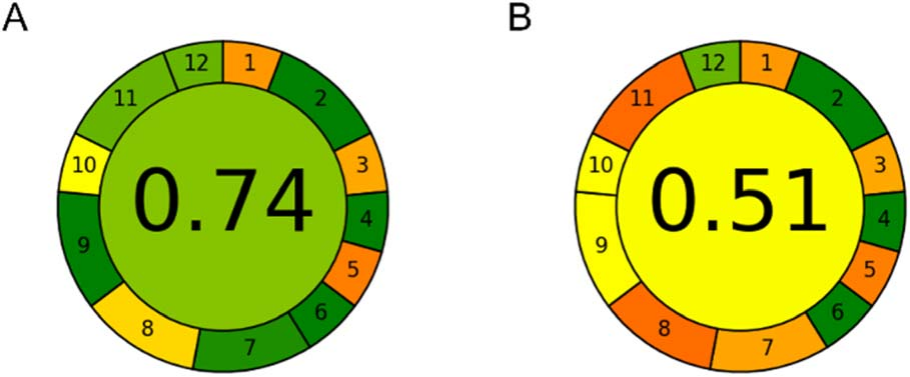
AGREE assessment results: (A) CE method and (B) HPLC method.

The environmental impact of both methods was assessed using AGREE software.^19^ Custom weights were attributed to sample preparation-related parameters (weight 1 – equal conditions in both cases) and technique-related parameters (weight 2). Exact inputs for CE and HPLC methods for all AGREE criteria are given in ESI Table S1.† Detailed calculation of AGREE scores is given in ESI Table S2.† The calculated AGREE scores are presented in Fig. 6.

It can be seen that the CE method achieved an AGREE score of 0.74 compared to an AGREE score of 0.51 in the HPLC method. Thus, the CE method is significantly more environmentally friendly. The main observed advantages of the CE method over the HPLC method were in the amount of waste per analysis (0.15 mL of the background electrolyte as opposed to 16 mL of the mobile phase), sample throughput (7 samples per hour instead of 3), energy consumption (0.015 kW h at most), and reduced use of toxic reagents (0.01 mL of MeOH present in the sample, instead of 9.5 mL in the HPLC mobile phase for the duration of each analytical run). Both methods require an equally small sample amount – only 20 μL. However, CE injects only a few nanolitres of the sample and can therefore inject the same sample many times if needed. As it can be seen, CE has weak points in criteria 1, 3 and 5. This is due to the external sampling and sample preparation which is not miniaturized as well as the at-line position of the instrument. Besides this, the HPLC method has additional weak points in criteria 8, 9 and 11 due to the relatively higher amounts of waste generated and toxic chemicals utilized while also having lower sample throughput.

## Conclusion

In this work, novel CE and HPLC methods for the determination of ALP in pharmaceutical dosage forms were developed, validated according to ICH guidelines and applied to real pharmaceutical samples. In addition, methods were cross-validated, and it was shown that there is no significant difference in the analytical performances of both methods. The greenness assessment was done on both methods showing that the CE method is significantly greener concerning waste production, sample throughput and energy consumption. It is worth noting that this is the first-ever CE method for the analysis of ALP in any sample matrix and as such is a valuable contribution to the analysis of ALP in general. In the end, due to equally reliable analytical performance and greener analysis, CE should be considered as an alternative technique to HPLC in the analysis of ALP pharmaceutical dosage forms.

## Data availability

The data supporting this article have been included as part of the ESI.†

## Author contributions

Zvonimir Mlinarić: conceptualization, methodology, investigation, formal analysis, data curation, validation, visualization, writing – original draft. Lu Turković: conceptualization, methodology, investigation, formal analysis, data curation, validation, visualization, writing – reviewing and editing. Ivor Babić: methodology, investigation, data curation. Tajana Silovski: validation, writing – reviewing and editing, resources. Nina Kočevar Glavač: validation, writing – reviewing and editing. Miranda Sertić: conceptualization, methodology, validation, writing – reviewing and editing, resources, supervision, project administration, funding acquisition.

## Conflicts of interest

T. S. received speaker honoraria from Eli Lilly, Pfizer and Novartis and conference reimbursement from Novartis and Pfizer; is a consultant of Novartis and Pfizer and is a co-investigator in Novartis clinical study. Other authors declare no conflict of interest related to this study. The funders had no role in the design of the study; in the collection, analyses or interpretation of data; in the writing of the manuscript, or in the decision to publish the results.

## Supplementary Material

RA-014-D4RA05715E-s001
