# Supplementary material for: Development, cross-validation and greenness assessment of capillary electrophoresis method for determination of ALP in pharmaceutical dosage forms – an alternative to liquid chromatography
Source: RSC Adv. 2024 Oct 18;14(45):32876–82. doi: 10.1039/d4ra05715e (PMC11487321; doi:10.1039/d4ra05715e)
Supplement: RA-014-D4RA05715E-s001 [file RA-014-D4RA05715E-s001.pdf]

Supplementary Information (SI) for RSC Advances.
This journal is © The Royal Society of Chemistry 2024

Supplementary information

Development, cross-validation and greenness assessment of capillary electrophoresis method for determination of alpelisib in pharmaceutical dosage forms – an alternative to liquid chromatography

Zvonimir Mlinarić [a], Lu Turković [a], Ivor Babić [a], Tajana Silovski [b,c], Nina Kočevar Glavač [d], Miranda Sertić [a]

[a] University of Zagreb Faculty of Pharmacy and Biochemistry, Department of Pharmaceutical Analysis, Ante Kovačića 1, 10000 Zagreb, Croatia

[b] University Hospital Centre Zagreb, Department of Oncology, Kišpatićeva 12, 10000 Zagreb, Croatia

[c] University of Zagreb School of Medicine, Šalata 2, 10000 Zagreb, Croatia

[d] University of Ljubljana, Faculty of Pharmacy, Department of Pharmaceutical Biology, Aškerčeva cesta 7, 1000, Ljubljana, Slovenia

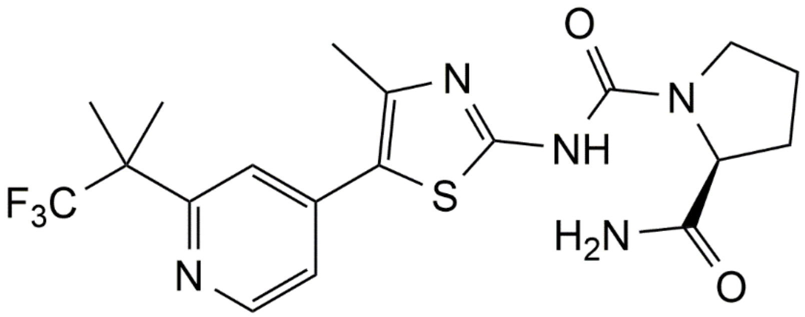

Figure S1. Chemical structure of alpelisib. Drawn by ChemDraw Professional 15.0 (PerkinElmer, Shelton, CT, USA)

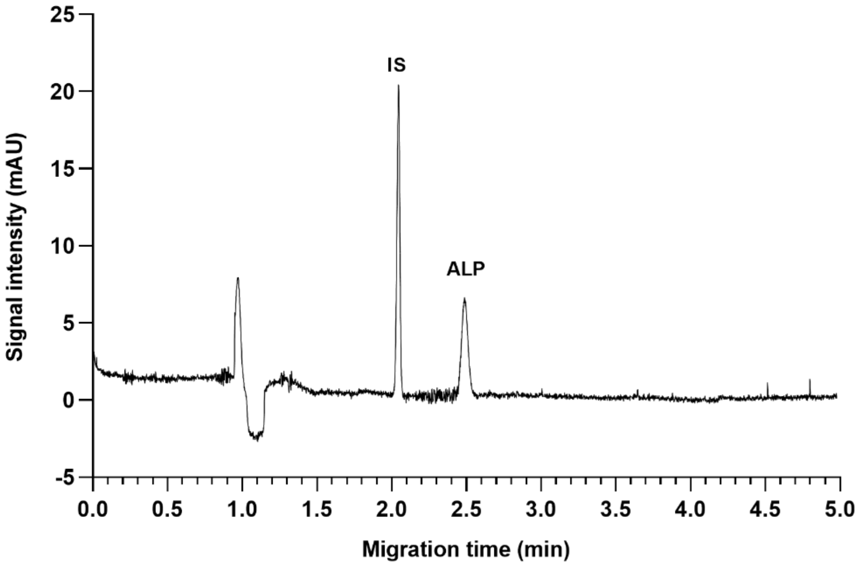

Figure S2. MEKC electropherogram of ALP and IS, 25 mM borate at pH 9.3, 20 mM SDS, 30 kV, 30 °C

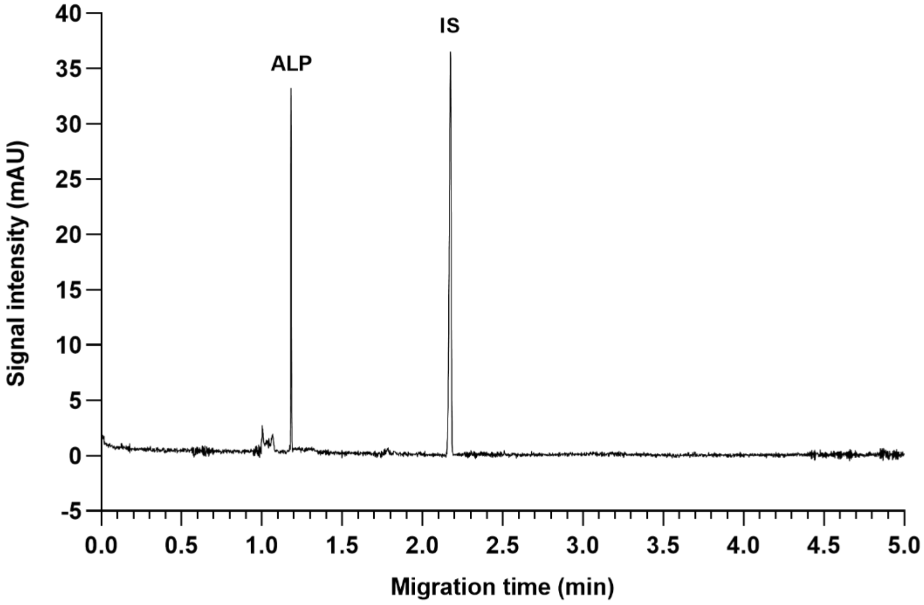

Figure S3. Electropherogram of ALP and IS, 25 mM borate at pH 9.3, 10% MeOH, 30 mM borate, 30 °C, 30 kV

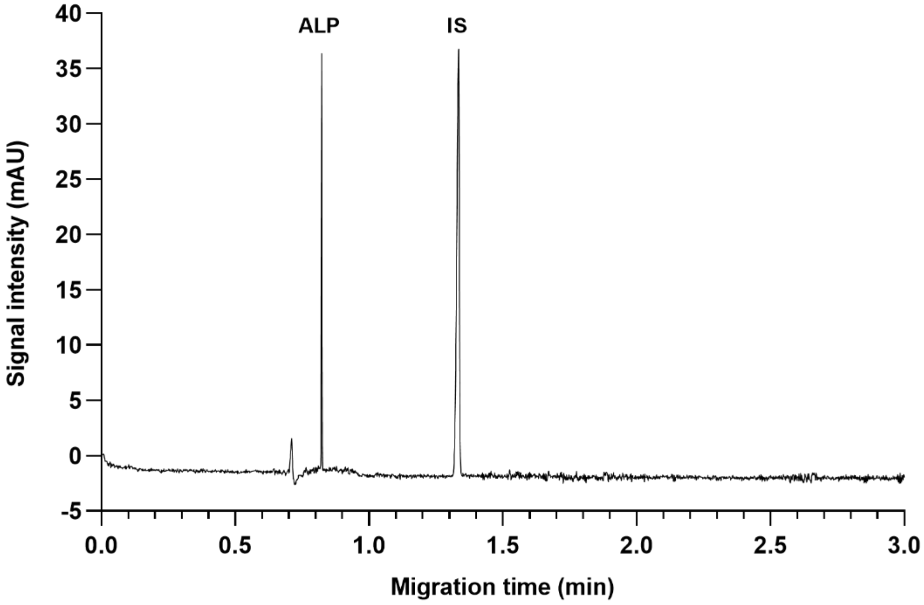

Figure S4. Electropherogram of ALP pharmaceutical dosage form with added IS at 50 µg/mL, 25 mM borate at pH 9.3, 30 kV, 30 °C

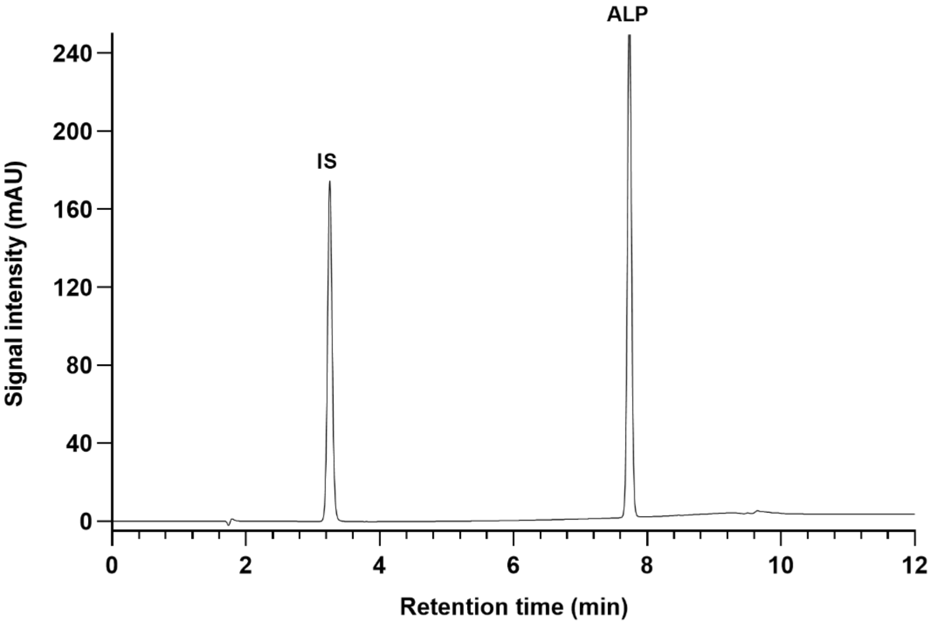

Figure S5. Chromatogram of ALP pharmaceutical dosage form with added IS at 50 µg/mL, detection at 315 nm

Table S1. Inputs for calculation of AGREE scores for CE and LC methods

Principle	Weight	CE	LC
Sampling	1	External sample preparation (reduced steps)	External sample preparation (reduced steps)
Amount of sample (g or mL)	2	0.02	0.02
Positioning of analytical device	1	at-line	at-line
Major steps	1	3 or fewer	3 or fewer
Automation; sample preparation	1	semi-automatic, not miniaturized	semi-automatic, not miniaturized
Derivatization	1	no	no
Amount of waste (g or mL)	2	0.15 mL	16 mL
Number of analytes, samples per hour	2	1 analyte, 7 samples per hour	1 analyte, 3 samples per hour
Energy	2	0,015 kWh	LC
Bio-based reagents	1	Some are bio-based	Some are bio-based
Use of toxic reagents	2	Yes, 0.01 mL	Yes, 9.5 mL
Threats	1	Highly flammable	Highly flammable

Table S2. AGREE scores for individual greenness criteria for CE and HPLC methods

Principle	Weight (w_i)	CE score (s_i)	LC score (s_i)
Sampling	1	0.30	0.30
Amount of sample (g or mL)	2	1.00	1.00
Positioning of analytical device	1	0.33	0.33
Major steps	1	1.00	1.00
Automation; sample preparation	1	0.25	0.25
Derivatization	1	1.00	1.00
Amount of waste (g or mL)	2	0.9488	0.3231
Number of analytes, samples per hour	2	0.4210	0.2152
Energy	2	1.00	0.50
Bio-based reagents	1	0.50	0.50
Use of toxic reagents	2	0.80	0.2108
Threats	1	0.80	0.80

AGREE score can be calculated according to the following equation where w_i is the weight factor for the greenness principle i with a corresponding individual score s_i.

$$AGREE\ score = \frac{\sum_{i=1}^{12} w_i s_i}{\sum_{i=1}^{12} w_i}$$

AGREE scores for CE and HPLC methods are then calculated as follows:

$$AGREE\ score\ (CE) = \frac{\sum_{i=1}^{12} w_i s_i}{\sum_{i=1}^{12} w_i} = \frac{12.5196}{17} = 0.7364$$

$$AGREE\ score\ (HPLC) = \frac{\sum_{i=1}^{12} w_i s_i}{\sum_{i=1}^{12} w_i} = \frac{8.6782}{17} = 0.5105$$
